# Accelerated *Varroa destructor* population growth in honey bee (*Apis mellifera*) colonies is associated with visitation from non-natal bees

**DOI:** 10.1038/s41598-021-86558-8

**Published:** 2021-03-29

**Authors:** Kelly Kulhanek, Andrew Garavito, Dennis vanEngelsdorp

**Affiliations:** 1 Department of Entomology, Washington State University, Pullman, WA USA; 2 Department of Entomology, University of Maryland, College Park, MD USA

**Keywords:** Agroecology, Ecosystem services, Entomology

## Abstract

A leading cause of managed honey bee colony mortality in the US, *Varroa destructor* populations typically exceed damaging levels in the fall. One explanation for rapid population increases is migration of mite carrying bees between colonies. Here, the degree to which bees from high and low mite donor colonies move between apiaries, and the effect visitation has on *Varroa* populations was monitored. More bees from low mite colonies (n = 37) were detected in receiver apiaries than bees from high mite colonies (n = 10, *p* < *0.001*). Receiver colony *Varroa* population growth was associated with visitation by non-natal bees (*p* = *0.03*), but not high mite bees alone (*p* = *0.19*). Finally, colonies lacking robbing screens experienced faster *Varroa* population growth than screened neighbors (*p* = *0.01*). Results indicate visiting non-natal bees may vector mites to receiver colonies. These results do not support the current two leading theories regarding mite immigration – the “mite bomb” theory (bees from high mite colonies emigrating to collapsing colonies), or the “robbing” theory (natal robbing bees return home with mites from collapsing colonies). Potential host-parasite effects to bee behavior, as well as important management implications both for *Varroa* treatment regimens and breeding *Varroa* resistant bees are discussed.

## Introduction

Honey bee provided pollination services to US crops are valued at over $14 billion^1^. Crop yields are influenced by the density and quality of honey bee colonies placed in fields, groves, and orchards^2–6^. High rates of honey bee colony mortality threaten pollinator dependent crop production^7,8^. Many colony health stressors exist^7,9,10^; the parasitic mite *Varroa destructor*, however, has garnered special attention as a driver of losses^11–13^. *Varroa* is particularly detrimental to colony health because of the damage it causes while feeding^14,15^, and by vectoring viruses that weaken the colony^16,17^. Over 50% of samples collected in the US over the critical months of August-November have mite levels well above the recommended management threshold of 3 mites/ 100 bees^18^. This is indicative of two non-mutually exclusive issues: beekeepers underestimate *Varroa* infestation levels in their operations, and/or beekeepers’ management of infestations are failing.

Management surveys show that on average between 2010 and 2018, 53% of backyard beekeepers (beekeepers with 1–50 colonies) did not perform *Varroa* management. These beekeepers experience winter losses 12.5 percentage points higher than their *Varroa*-managing counterparts (51.3% compared to 38.8%, respectively)^9,19^. Lower colony loss rates are correlated with use of common Varroacide products; an expected result considering the robust modeling and field trials that demonstrate the benefits of managing mite populations^13,17,20,21^. Beekeepers who identity as “management free” tend to believe honey bees perform best when left alone and so do not manage for *Varroa*^22^. Untreated colonies in a landscape crowded with beekeepers, however, can represent a real risk of horizontal transmission of mites, putting nearby treated colonies at risk.

Even among beekeepers who do monitor and treat for *Varroa*, infestation loads in the fall are often difficult to control. Long term studies on *Varroa* population growth over time, and mathematical models of *Varroa* population growth suggest that colonies can survive for three years with no *Varroa* control in temperate climates^23–25^. In reality, most beekeepers need to use multiple *Varroa* treatments per year to keep levels below damaging thresholds^19,21^. Further, longitudinal monitoring of *Varroa* loads in multiple apiaries across the US found that even after treatments, *Varroa* population growth rates often far exceed predicted levels based on *Varroa* reproduction rates alone^25^. This suggests that mites are immigrating into colonies from an outside source, most likely nearby colonies.

Bees often drift between colonies, representing a potential route for *Varroa* transmission^26,27^. Crowding of colonies within apiaries and throughout the landscape results in increased *Varroa* infestations as bees are more likely to move between colonies^28,29^. Colonies can also acquire mites when their bees rob other mite infested colonies, and/or when non-natal bees drift from other colonies^30,31^. Bees' propensity to drift into non-natal colonies increases when the bees have been parasitized by mites^32^. “Robbing” bees can also enter non-natal, and often weakly guarded colonies, in order to rob honey during periods of food scarcity^29^. Robbing behavior is especially prevalent in the fall when colonies with unchecked *Varroa* infestations start to collapse. These weakened colonies with elevated *Varroa* loads are robbed by nearby healthy colonies whose robbing bees may pick up mites and bring them back to their own colony^33,34^. Late fall is a critical time period for beekeepers as they prepare their bees for winter, ensuring food stores are adequate, mite loads are low, and colonies are healthy^35^. Re-infestation of *Varroa* during this period can undo the effects of successful management. Understanding the mechanism by which this late fall inter-apiary mite transmission occurs is critical in developing best management practices to help mitigate colony health damage.

Past studies have attempted to document and explain inter-apiary mite transmission, but understanding its directionality requires the difficult task of tracking individual bees across the landscape. Most prior work has tracked changes in mite loads in situations hypothesized to increase drift and *Varroa* transmission without tracking bees^28–31^. Some studies have tracked bees, but only on a small number of colonies or within one apiary^33,34,36^. Here, 32 colonies in eight receiver apiaries up to 1.6 km from a central donor apiary were used to reflect realistic landscape level situations. The degree to which bees moved between apiaries from high and low mite donor colonies was tracked, and changes in *Varroa* loads were monitored. Additionally, robbing screens were used to keep out non-natal bees from 50% of receiver colonies. This novel application of an existing beekeeping tool provided important clues about the directionality of inter-colonial mite transmission.

Two inter-apiary mite transmission hypotheses were tested.

1. The “mite bomb” hypothesis: If mites were primarily transmitted by bees from high mite donor colonies, the following was expected:
  The majority of visitors to receiver colonies should be high mite donor bees
  Mite loads in visited colonies should increase more than in non-visited colonies
  Colonies with robbing screens should exhibit smaller increases in mite loads than their unscreened counterparts, as screens would inhibit non-natal high mite bees from entering
2. The “robbing” hypothesis: if mites were primarily transmitted by bees from healthy receiver colonies robbing out high mite donor colonies, the following was expected:
  There should be no difference in the frequency of visitations to receiver colonies by high and low mite donor bees
  Increases in mite loads in receiver colonies should not be associated with donor bee visitation
  Colonies with robbing screens should not exhibit different *Varroa* population growth compared to unscreened colonies, as natal bees bringing home mites after robbing would not be deterred by the screens

## Methods

### Apiaries

Two types of apiaries were established for this study: eight receiver apiaries and one donor apiary. Receiver apiaries consisted of four colonies each, housed in either a single 10 frame Langstroth deep brood box (n = 28) or one 10 frame Langstroth deep and one 10 frame Langstroth medium brood box (n = 4). All receiver colonies were established from splits with new queens in August 2019 to equalize colony strength and facilitate movement into the experimental location. Each receiver colony was made of seven frames of adult bees, three frames of brood, and two frames of honey. Receiver colonies were moved into the experimental location on August 30th, and received a *Varroa* treatment (Mite Away Quick Strips, NOD Apiary Products, Alberta, CAN) from September 18th to September 24th to ensure low initial mite loads.

The donor apiary consisted of two high mite colonies (*Varroa* load > 3 mites/100 bees) and two low mite colonies (*Varroa* load < 1 mite/100 bees). Donor colonies were overwintered and selected based on results of alcohol washes performed in August. Low mite colonies received a half dose formic acid treatment before the experiment began (September 18th to September 24th) to ensure a low initial mite load. The donor apiary was established at the experimental location on September 27th. Low mite colonies received a second half dose formic acid treatment on October 3rd, to combat any *Varroa* increases they had incurred from their close proximity to the high mite colonies, as substantial drift within the donor apiary was assumed. This additional mite treatment means that low mite donor colony mite population growth rates cannot be compared to receiver colony mite population growth rates.

**Figure 1:**
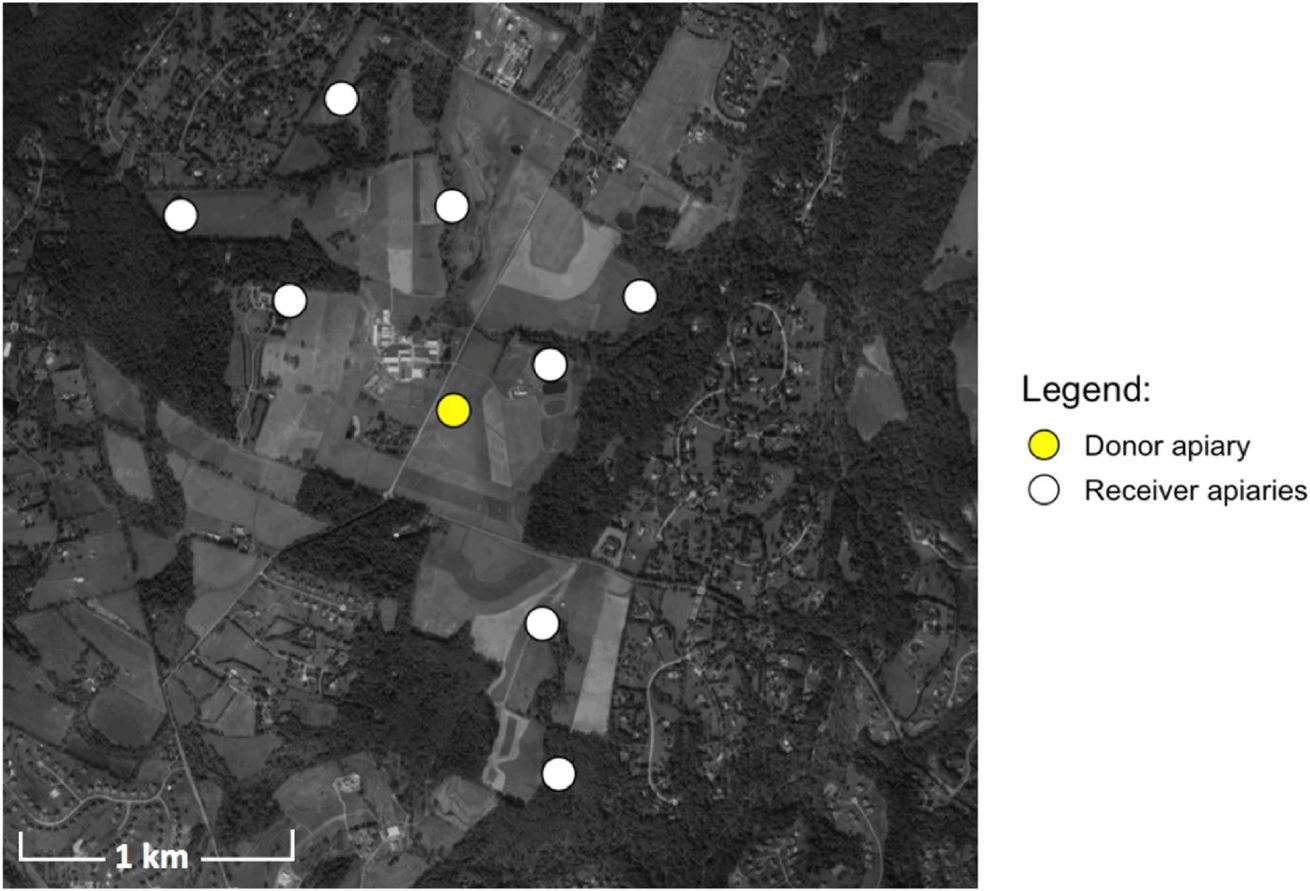
Map of donor and receiver apiary locations. Map produced with R version 3.3.3 (https://www.r-project.org/).

All apiaries were placed at the Central Maryland Research and Education Center located at Clarksville, Maryland. The donor apiary was placed near the geographic center of the farm. Four receiver apiaries were placed approximately 0.8 km (0.5 mi) from the donor apiary. Four additional receiver apiaries were placed approximately 1.6 km (1 mile) from the donor apiary (Fig. 1).

### Painting bees

To achieve maximum possible detection of bee movement between apiaries, as many bees in the donor apiary were painted as possible. A painting method that would not interrupt the bee or *Varroa* brood cycles was necessary, so painting cohorts of emerging bees in the lab was rejected. Most methods of painting bees in the field involve placing a marker over the colony entrance, but this method paints both foragers native to that colony and any robbing or non-natal bees who pass through the entrance^37^. To ensure only bees originating from each donor colony were painted, the following method was developed.

**Figure 2:**
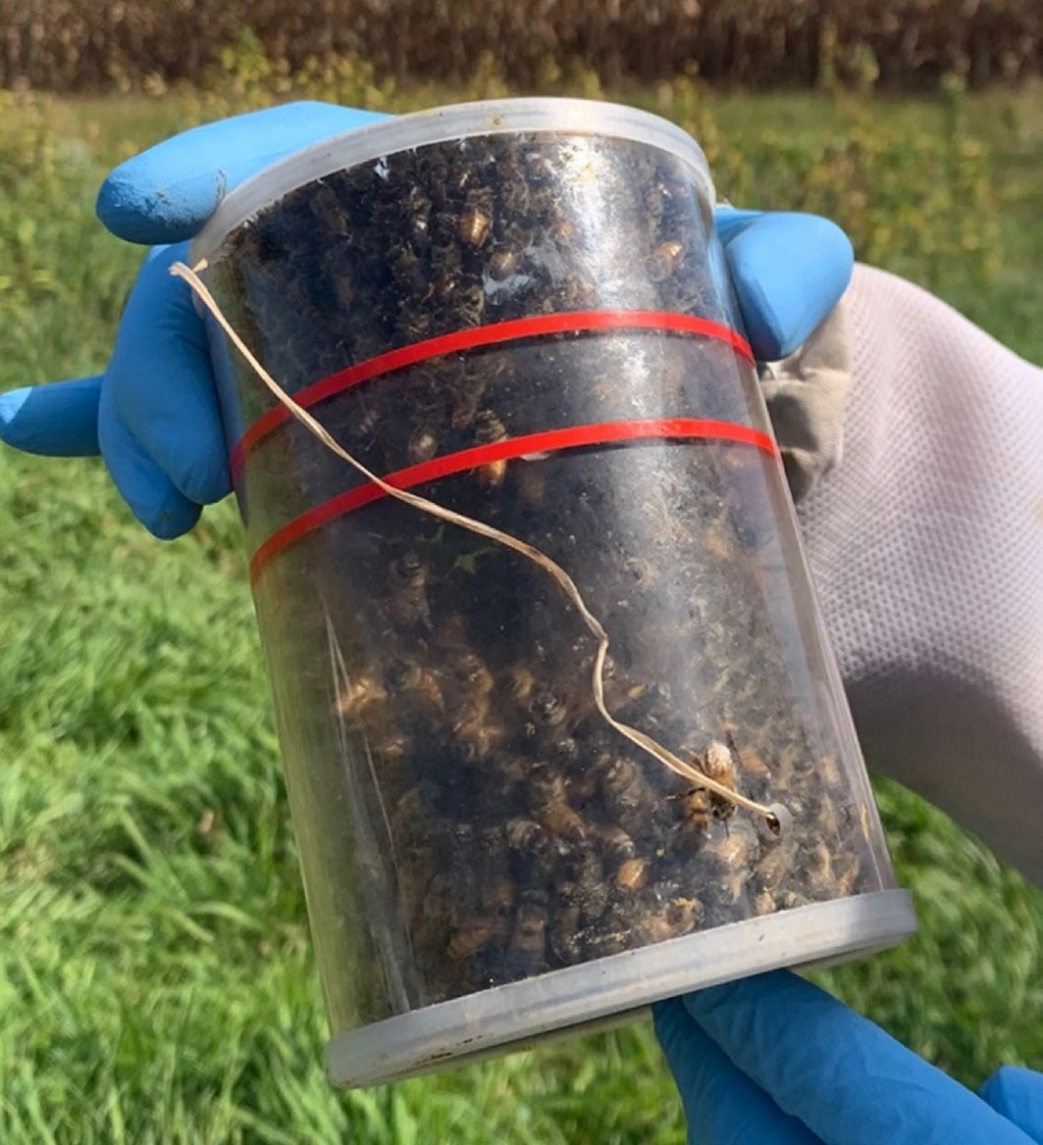
Plastic cylinder with ~ 500 bees before CO_2_ was injected.

**Figure 3:**
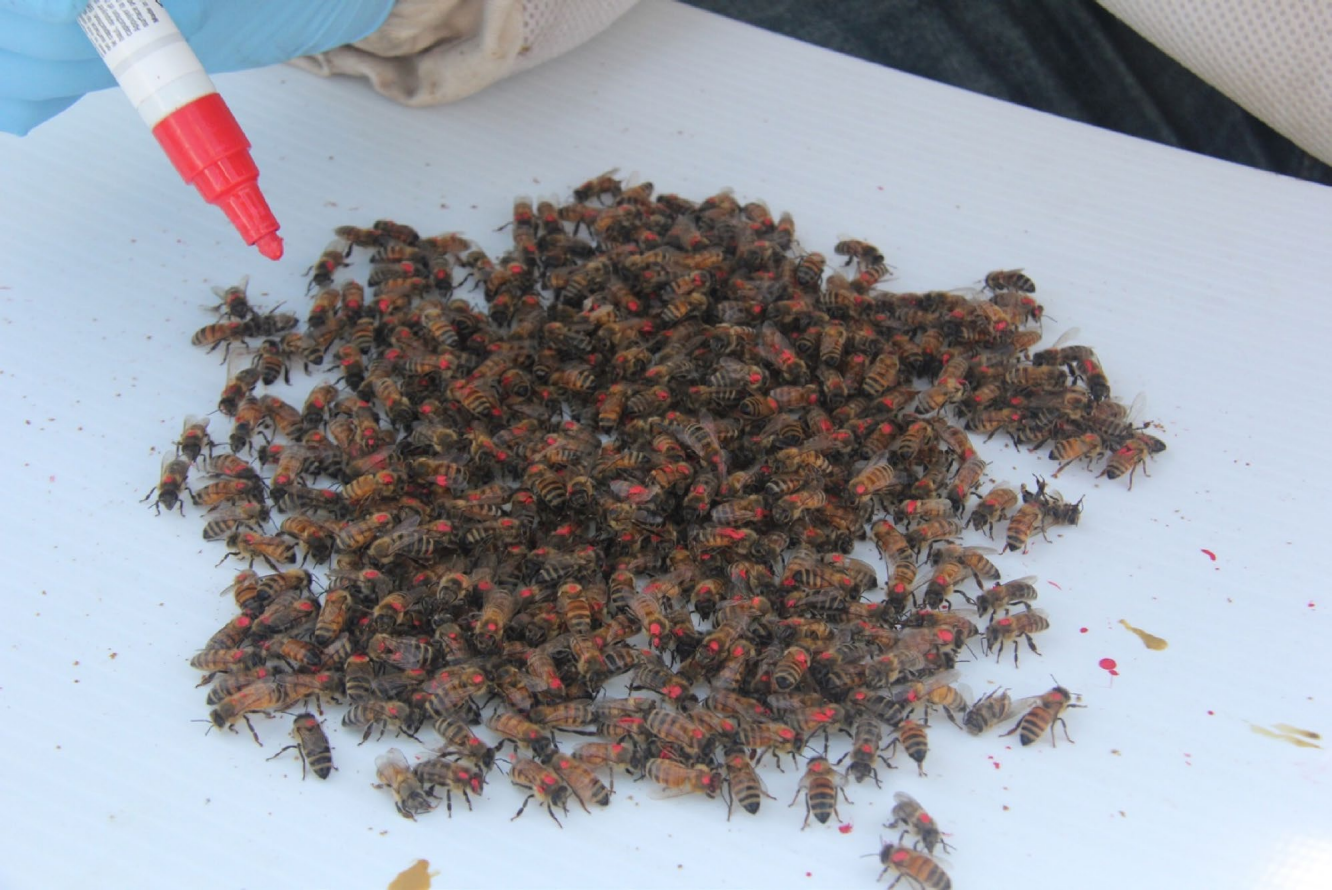
A batch of freshly painted bees on a flat surface. Here IPM sticky boards were used.

All frames of adult bees were shaken into a plastic tub one at a time, covering the tub with its lid in between each frame. This resulted in containing the majority (~ 90%) of the adult bee population in the tub. The lid of the tub was then lifted just enough to scoop ~ 500 bees into a small plastic cylinder (Fig. 2, CO_2_ *Varroa* Tester: Logar Beekeeping Equipment, logar-trade.com). These *Varroa* testers have a small hole where CO_2_ can be injected into the cylinder. The bees received CO_2_ until they became unconscious (~ 5–10 s), and were then poured out onto a flat surface for painting (Fig. 3). The bees regained consciousness after about 15 s, but were disoriented and remained still enough to paint for up to 10 min. High mite colony bees were painted red, and low mite colony bees were painted blue (Sharpie Oil-Based Paint Marker). This process was repeated eight times for each donor colony, resulting in ~ 4,000 painted bees per colony. Painting occurred the day the colonies were moved to the experimental location on September 27th, and again three weeks later on October 18th as an entire new brood cycle had emerged and the proportion of painted bees in the colony had decreased.

### Camera sensors

**Figure 4:**
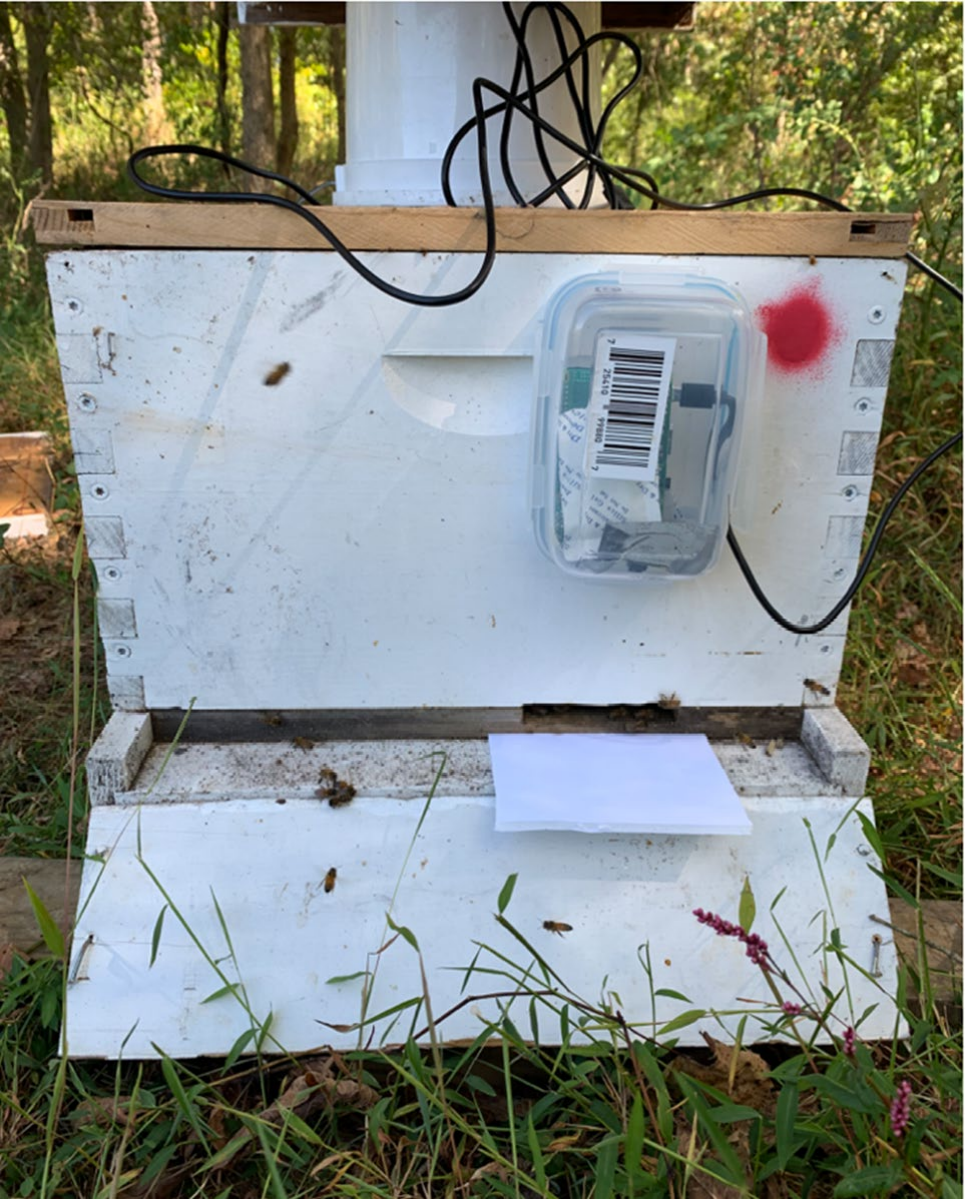
Receiver colony mounted with camera sensor.

In preliminary trials, it became evident that manually searching receiver colonies for painted bees was impractical. To overcome this hurdle, a camera sensor was developed to capture painted bees entering receiver colonies. A simple computer (Raspberry Pi 3B +) fitted with a camera module (Pi Camera 2) was programmed with OpenCV (Python 3) to detect user-specified colors. For this experiment, the RBG values associated with blue and red paint colors were used. A generous range of RBG values was used to account for variation in colors due to time of day, shade, or clouds. The cameras were programmed to capture a photo at 3 frames/second when they detected red or blue. Photos were saved with time and date stamps to help identify unique individuals. Colony entrances were reduced to limit the bees’ path of entry to within the camera’s field of view. White cardboard was mounted under cameras to provide a neutral backdrop. All 36 colonies in the experiment (donor and receiver) were mounted with a camera sensor from September 28th through November 10th (Fig. 4). Cameras were powered with 20,000 mAh high capacity power banks. These batteries were changed and recharged daily for the duration of the experiment.

**Figure 5:**
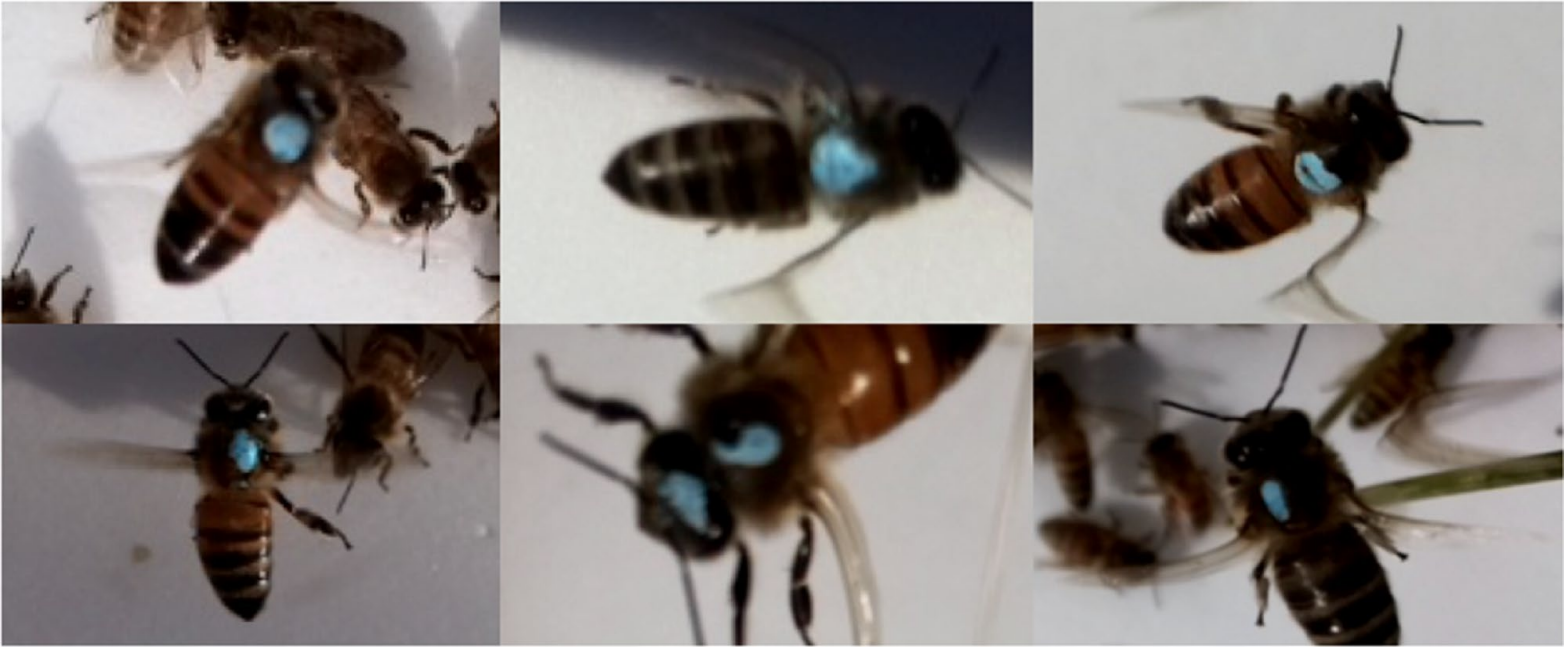
Examples of how unique paint markings can be used to tell individuals apart. Paint markings, in combination with the timing of photos, were used to determine unique visitations.

While cameras were mounted on all colonies, the robbing screens resulted in glare and interfered with the cameras’ field of vision, so the data presented here is from cameras mounted on colonies without robbing screens only. Additionally, many cameras took an exorbitant number of photos (between 20,000–60,000). Because the cameras were programmed with a generous range of RBG values to not miss any detections of painted bees in varying light, occasionally other colored objects in a camera’s field of vision (*e.g.* grass, fallen persimmons, etc.) appeared blue or red and triggered photo capture. To eliminate irrelevant photos, if a camera contained a set of over 1,000 photos taken in a short period of time, this was deemed an unlikely true detection and ignored. Sets of photos that contained fewer than 1,000 photos were checked for true detections of painted bees. Unique individuals could be discerned with reasonable confidence because their paint marks were typically distinctive (Fig. 5). Distinctive paint marks, in combination with visitations occurring on different days at different times, allowed the counting of the actual number of separate individual donor bees visiting receiver colonies. If there was any doubt as to whether photographed bees were unique individuals, the more conservative estimate that they were all one individual was used.

### Robbing screens

Robbing screens were placed on 50% of receiver colonies. In each receiver apiary, one colony in the middle and one on the end of the row received a robbing screen (Mann Lake, Hackensack, MN). Robbing screens are metal mesh that block the regular colony entrance, and have a separate hidden entrance at the top of the screen. Only bees that live in the screened colony learn the new entrance, and the reduced entrance is easier for natal bees to defend, so non-natal bees are deterred from entering. Whether the left most or right most colony was screened was chosen randomly, but screening both end colonies was avoided, as unpublished data suggests that end colonies are more susceptible to receiving visiting bees (vanEngelsdorp Pennsylvania State Inspection records). After the first screened colony was randomly chosen, a second colony not adjacent to the screened colony received a robbing screen.

### Monitoring and sampling

Donor and receiver colonies were monitored throughout the experiment for changes in *Varroa* load. An alcohol wash was performed at the beginning (September 24th) and the end (November 10th) of the experiment. Samples were checked for painted bees when processed, and no painted bees were found in receiver colony alcohol samples. Sticky boards placed under each receiver colony were changed and counted approximately every three days, dependent on weather. Each receiver colony was also manually checked for painted bees at the middle (October 23rd) and end (November 10th) of the experiment. Manual checks consisted of removing and visually inspecting every frame in each colony for painted bees.

**Figure 6:**
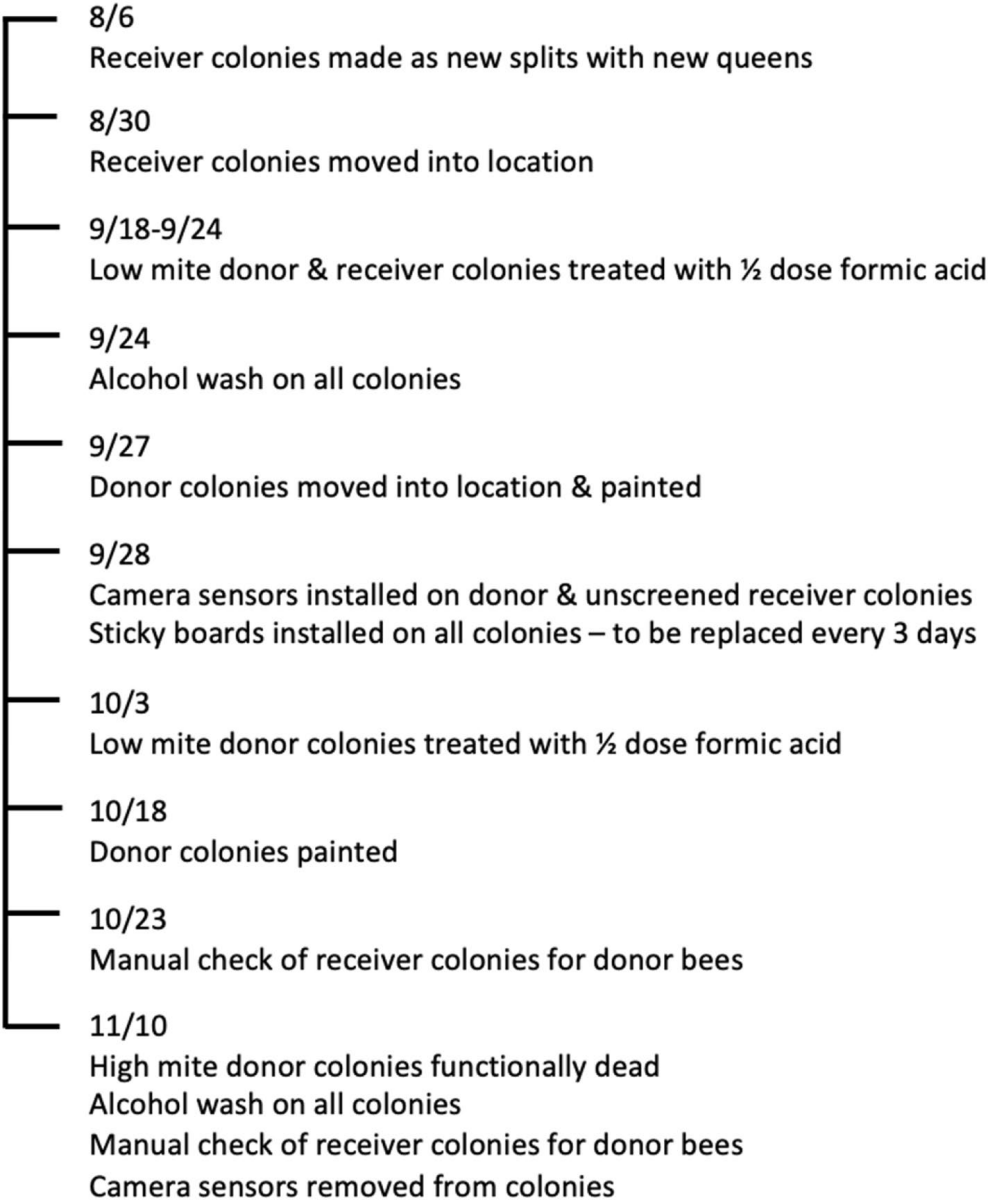
Timeline of study events including treatment, sampling, and colony preparation.

Donor colonies were checked once a week to monitor for paint retention and for colony size. The proportion of the population that was still painted was visually assessed as a percentage of the total adult bee population. When ~ 50% of the bees in a colony were unpainted (3 weeks and one brood cycle later), a second round of painting was performed. Colony size was assessed by the standard method of a frames of bees estimate by observing the top bars of each colony^38^. Receiver colonies were not assessed for colony size as they were identical at the start of the experiment. The goal of this study was to continue until either the high mite donor colonies collapsed (and the movement of bees from collapsing colonies could be tracked) or until the weather became so cold that bees no longer foraged regularly. Under these guidelines, the experiment was conducted from September 18th–November 10th (Fig. 6).

### Statistics

All statistical analyses were performed in R (version 3.3.3). Summary statistics are reported as mean ± SEM unless otherwise stated. Student’s t tests were used to check if *Varroa* loads and colony size were the same between treatment groups at the starting point of the experiment. Pearson’s Chi-squared test was used to check for differences in mite loads between colonies within the donor apiary, as well as in painted bee detections of each color in receiver colonies. Generalized mixed effects models with apiary as random effects were used to compare between groups at different time points (start and end or number of experimental weeks). Sampling time (start or end) was also included as a random effect to account for the pseudo replication of repeated measures of the same colonies at each time point. Models were eliminated in a stepwise fashion using ANOVAs until the simplest best fit model was identified. Spearman’s correlations were performed to check for correlations between the number of painted bees detected and the starting *Varroa* load.

## Results

### Donor colony adult bee populations

**Figure 7:**
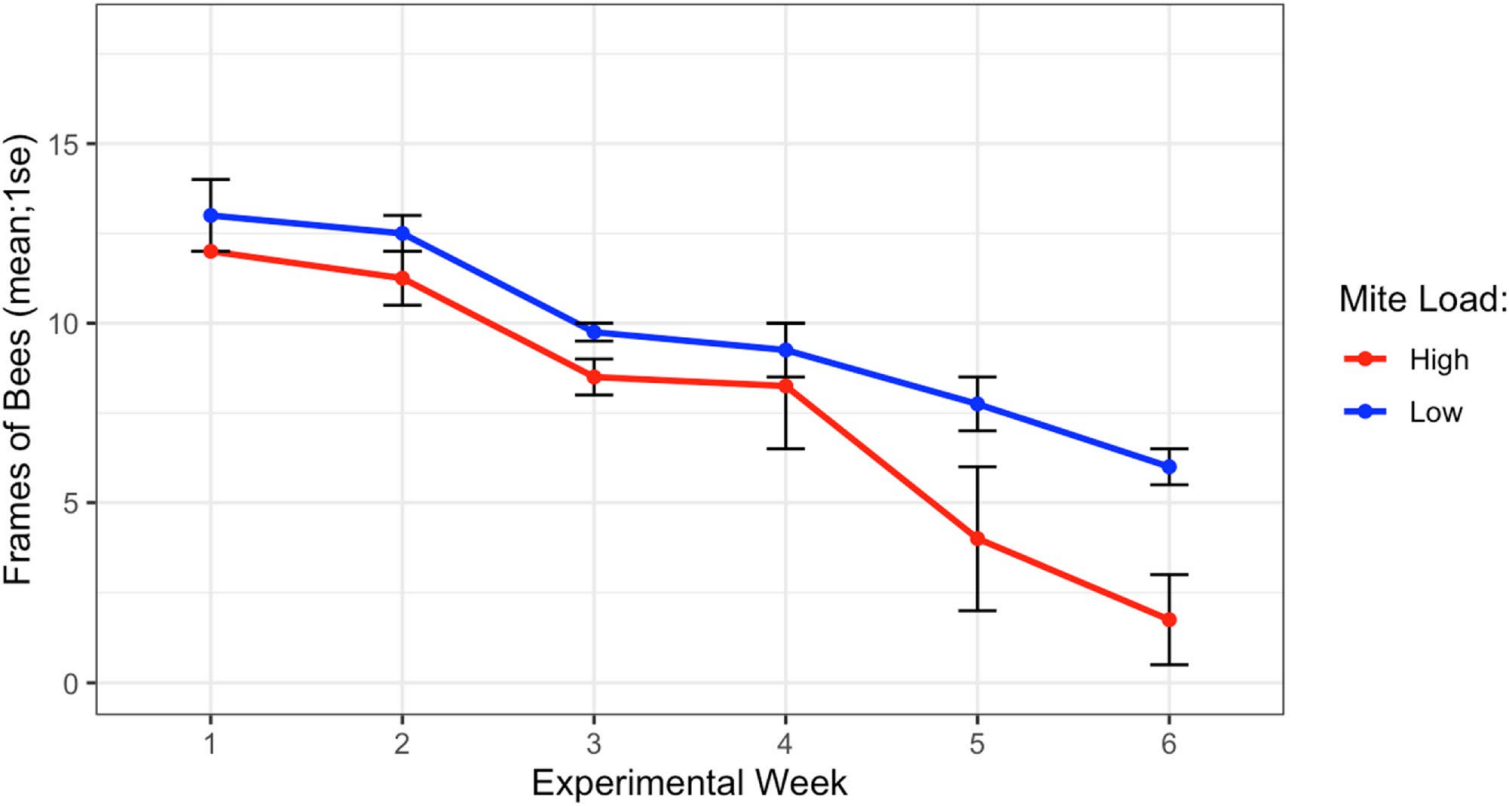
Population sizes of high and low mite donor colonies over each experimental week. Low mite colonies were significantly larger than high mite colonies over the duration of the study (glm ***p < 0.001).

The experiment began on Sep 28th, 2019 with all donor colonies at the same population size (Fig. 7, 12.5 ± 0.5 frames of bees, *t* = -1, *df* = 1, *p* = 0.5). At the end of the experiment on Nov 10th, 2019, high mite donor colonies had not rapidly collapsed as anticipated, but had steadily dwindled in population until functionally dead (< 1 frame of bees) and were smaller than the low mite donor colonies (high mite 1.75 ± 1.25 vs. low mite 6.0 ± 0.35 frames of bees, *F*_1_ = 12.49, *p* < 0.001). All 32 receiver colonies survived the entire length of the experiment and remained approximately the same size throughout.

### Detections of donor bees in receiver apiaries

**Figure 8:**
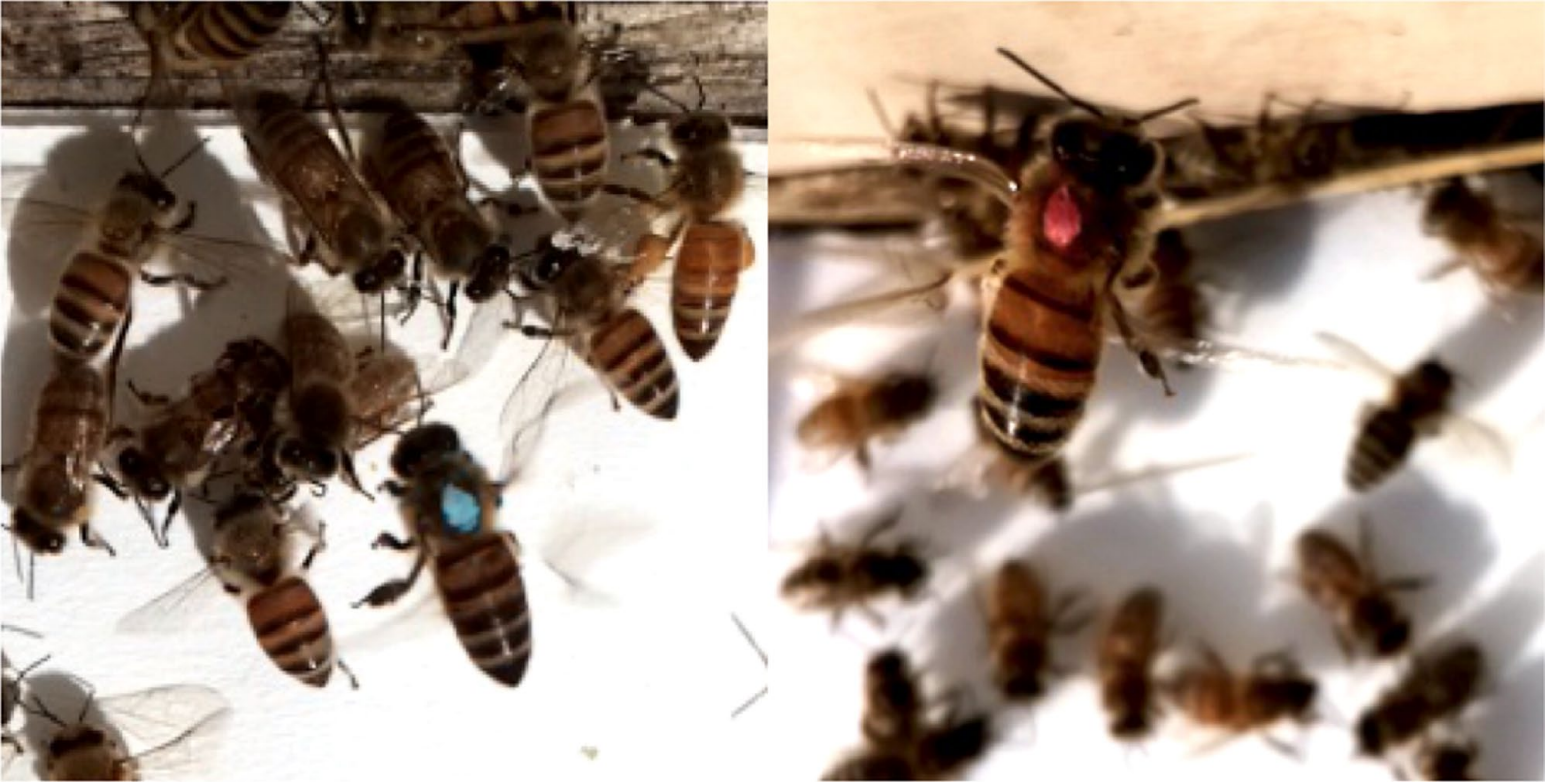
Photos of low mite (left) and high mite (right) bees taken at receiver colony entrances by camera sensors.

**Figure 9:**
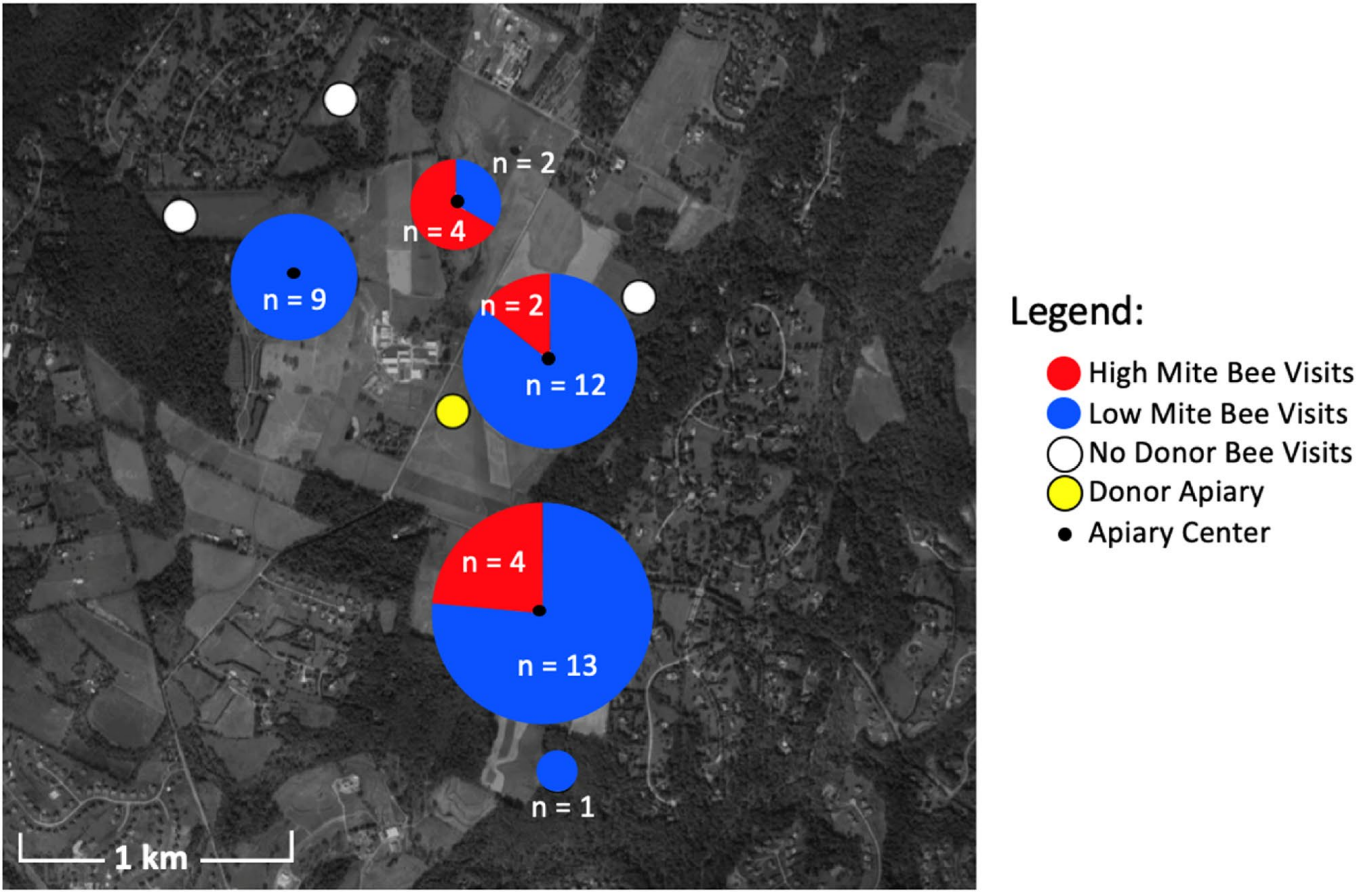
Location, number, and color of painted bee detections. Pie charts represent the number of high and low mite bees detected in each receiver apiary. Black dots represent the geographic center of each apiary. White circles are receiver apiaries where no painted bees were found, the yellow circle indicates the donor apiary. Map produced with R version 3.3.3 (https://www.r-project.org/).

In total, 47 unique bees from donor colonies were detected by 16 camera sensors on unscreened colonies (Fig. 8). Considering ~ 4,000 bees were painted in each of the 4 donor colonies at two time points, a total of 32,000 bees were painted. Thus the 47 bees detected represents a 0.15% recovery rate. Of the 47 detections, more low mite bees were detected (n = 37) than high mite bees (Fig. 9, n = 10, χ^2^ = 15.5, df = 1, p < 0.001). The number of detections of high or low mite bees was not correlated to high or low mite donor colony population size (high mite Spearman’s *r* = 0.37, *p* = 0.47, low mite Spearman’s *r* = -0.64, *p* = 0.17). Painted bees were detected in 62.5% (n = 5) of receiver apiaries and at 56.3% (n = 9) of non-screened receiver colonies.

There was substantial drift of bees between colonies within the donor apiary. Donor colonies were mounted with cameras, and the number of non-natal bees detected was higher than could be quantified (hundreds in each donor colony camera). Two manual checks performed of receiver colonies for painted bees did not result in any detections, suggesting painted bees did not permanently remain in non-natal colonies. With only one detection in apiaries placed at a further radius (1.6 km from donors), donor bees were much more likely to visit closer apiaries then further apiaries (χ^2^ = 43.1, *df* = 1, *p* < 0.001). The visited apiary at the further radius only received one visitor to one of the two unscreened colonies. In all other apiaries that received donor bee visitors, both unscreened colonies were visited.

### *Varroa* loads

**Figure 10:**
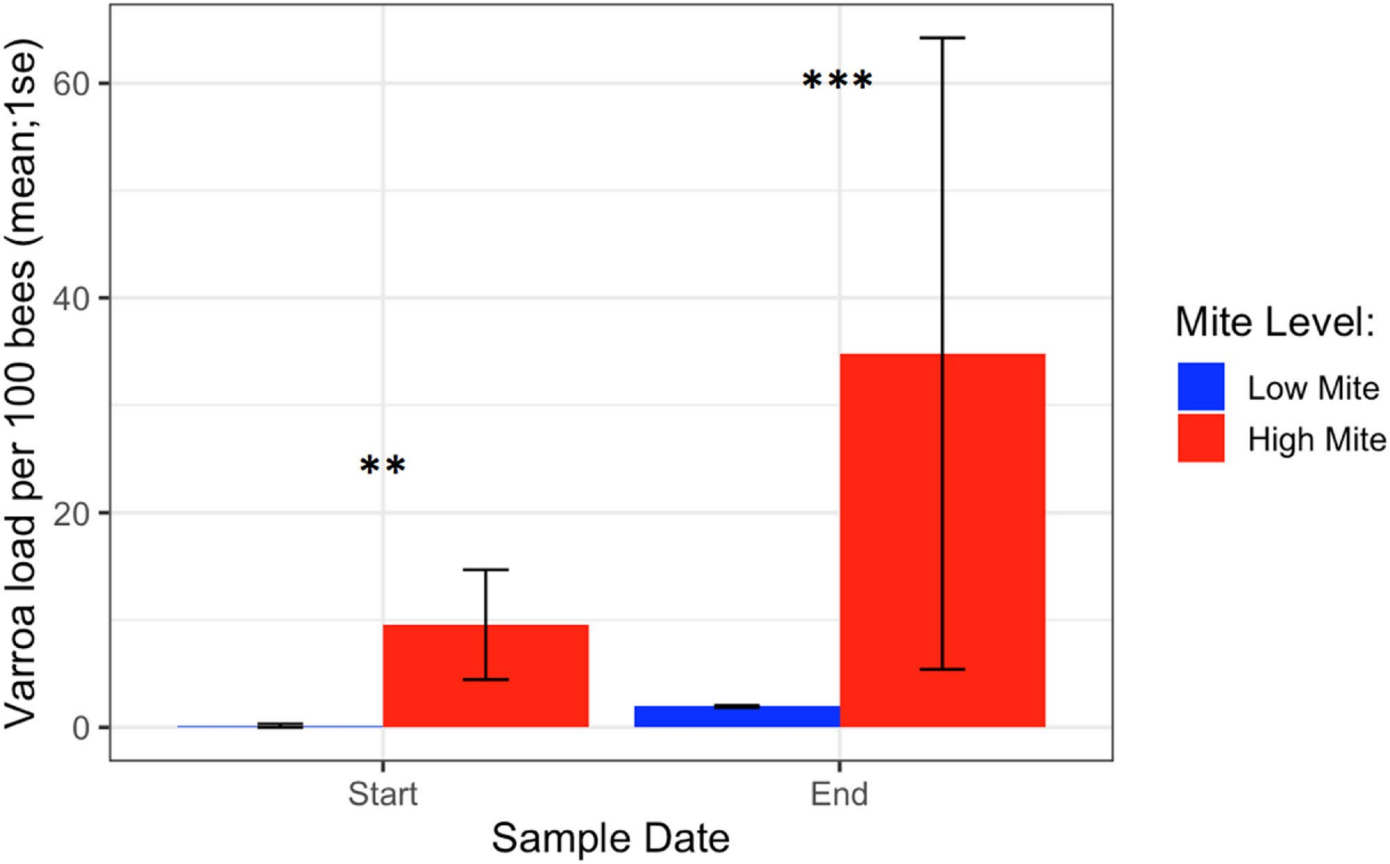
Varroa loads in low vs. high mite donor colonies at the start and end of the experiment. High mite colonies had significantly more mites than low mite colonies throughout the study (*χ*^*2*^ **p < 0.01, ***p < 0.001).

*Varroa* loads in low mite donor colonies remained low throughout the study (from 0.17 ± 0.17 to 1.95 ± 0.10 mites/ 100 bees), while *Varroa* loads in high mite donor colonies grew substantially (from 9.57 ± 5.12 to 34.8 ± 29.41 mites/ 100 bees). The two high mite colonies started with significantly different mite infestations (Supplementary Table 1, χ^2^ = 5.5, df = 1, p = 0.02), but their mite loads were always higher than in low mite colonies (Fig. 10, χ^2^ = 29.4, df = 1, p < 0.001). *Varroa* loads in all 32 receiver colonies increased over the duration of the study (from 0.91 ± 0.22 to 1.94 ± 0.32 mites/ 100 bees, p < 0.001). Receiver colonies also had laying queens and capped brood present throughout the study.

**Figure 11:**
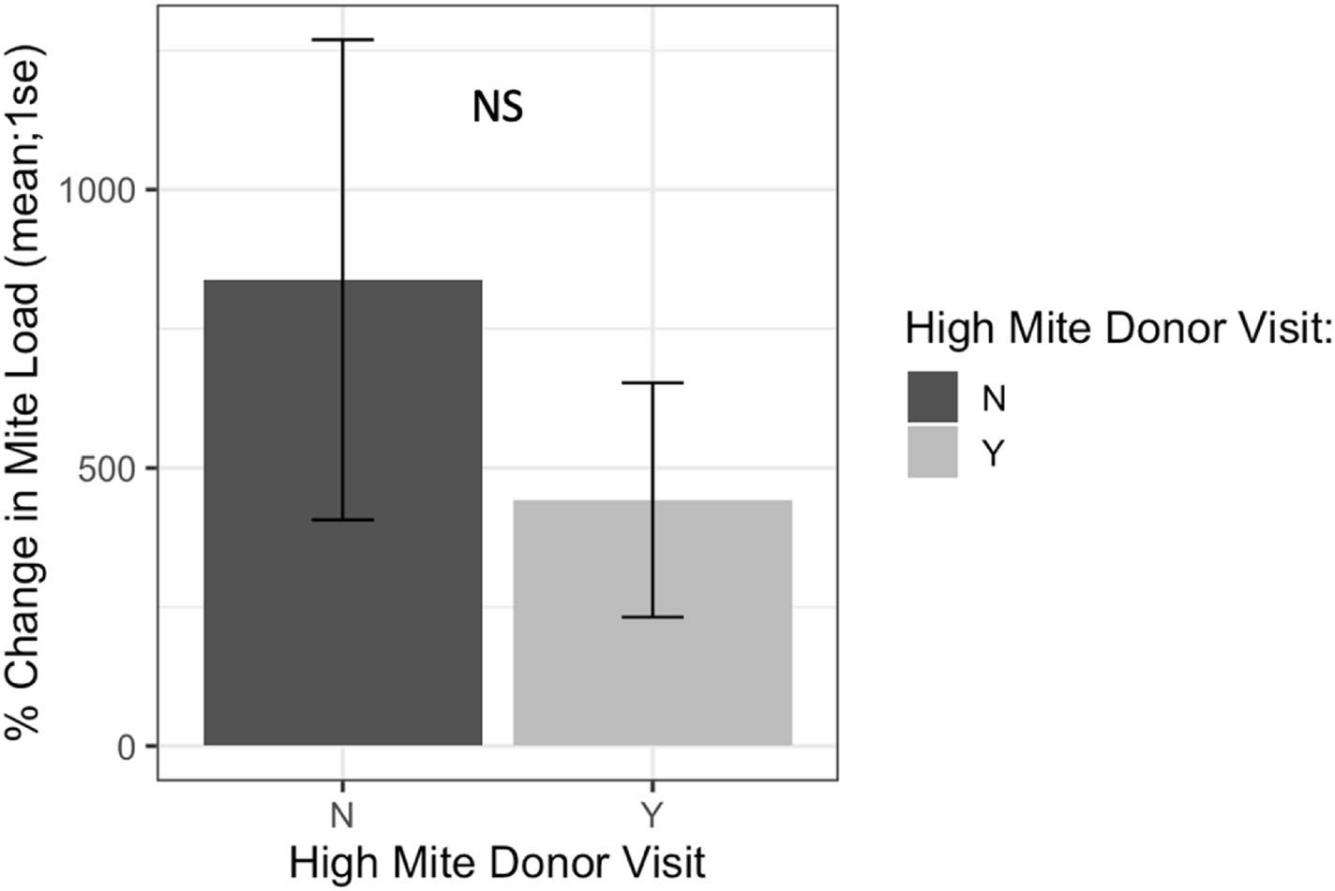
Percent change in Varroa loads in colonies that received high mite donor bees compared to colonies that did not receive high mite donor bees. There was no difference in percent change between colonies visited by high mite bees and unvisited colonies at the start or end of the study (*glm p* = 0.19).

Receiver colonies that were visited by high mite donor bees started the study with similar *Varroa* loads compared to colonies that were not visited by high mite donor bees (*t* = 0.80, *df* = 9.4, *p* = 0.45). Receiver colony mite population increase over the duration of the study was not affected by high mite donor bee visitation (Fig. 11, F1 = 1.42, *p* = 0.19).

**Figure 12:**
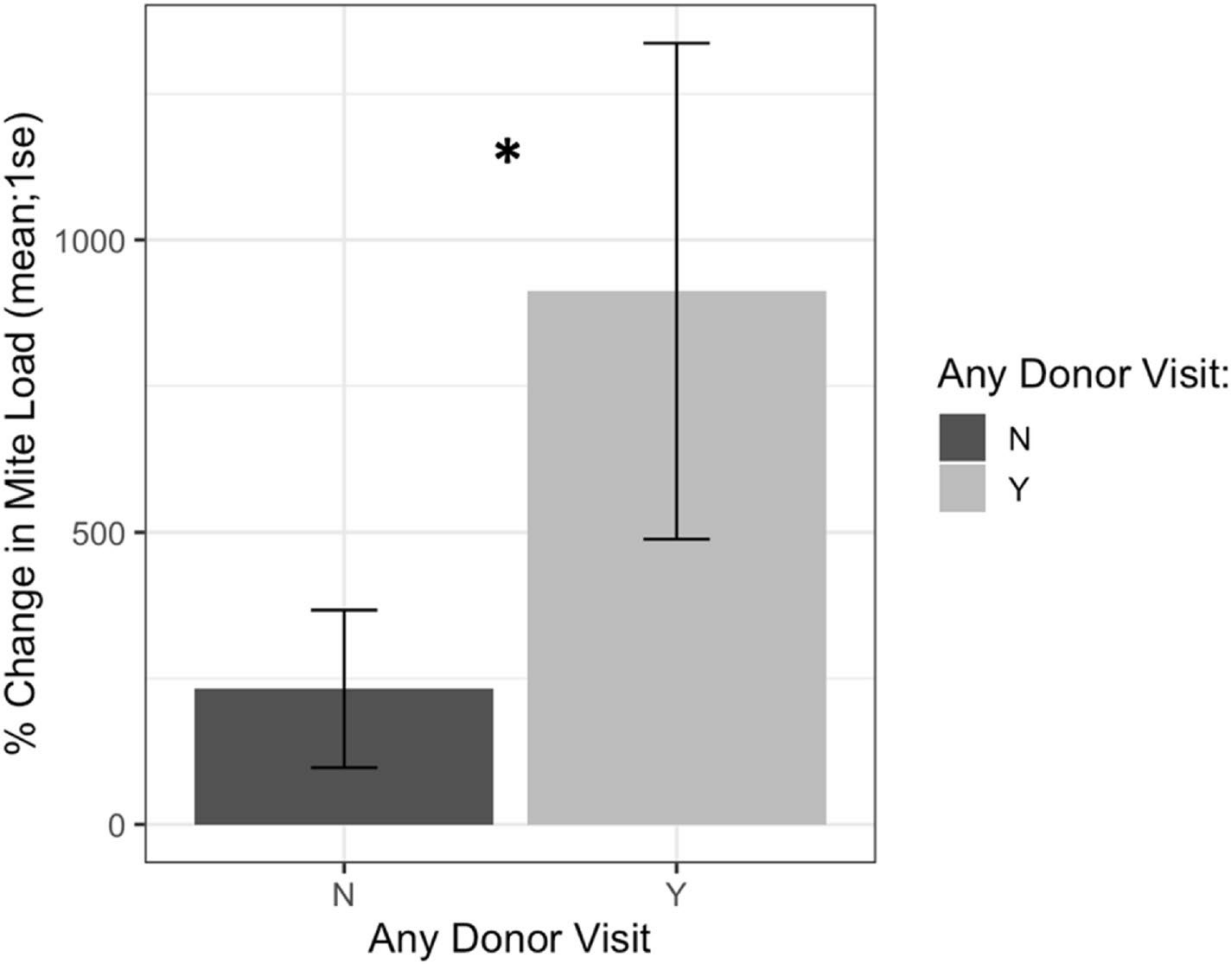
Percent change in Varroa load in colonies that received any donor bee (high or low mite) compared to colonies who did not receive any donor bee. Colonies that were visited by donor bees experienced significantly faster Varroa population growth than unvisited colonies (glm **p = 0.03).

**Figure 13:**
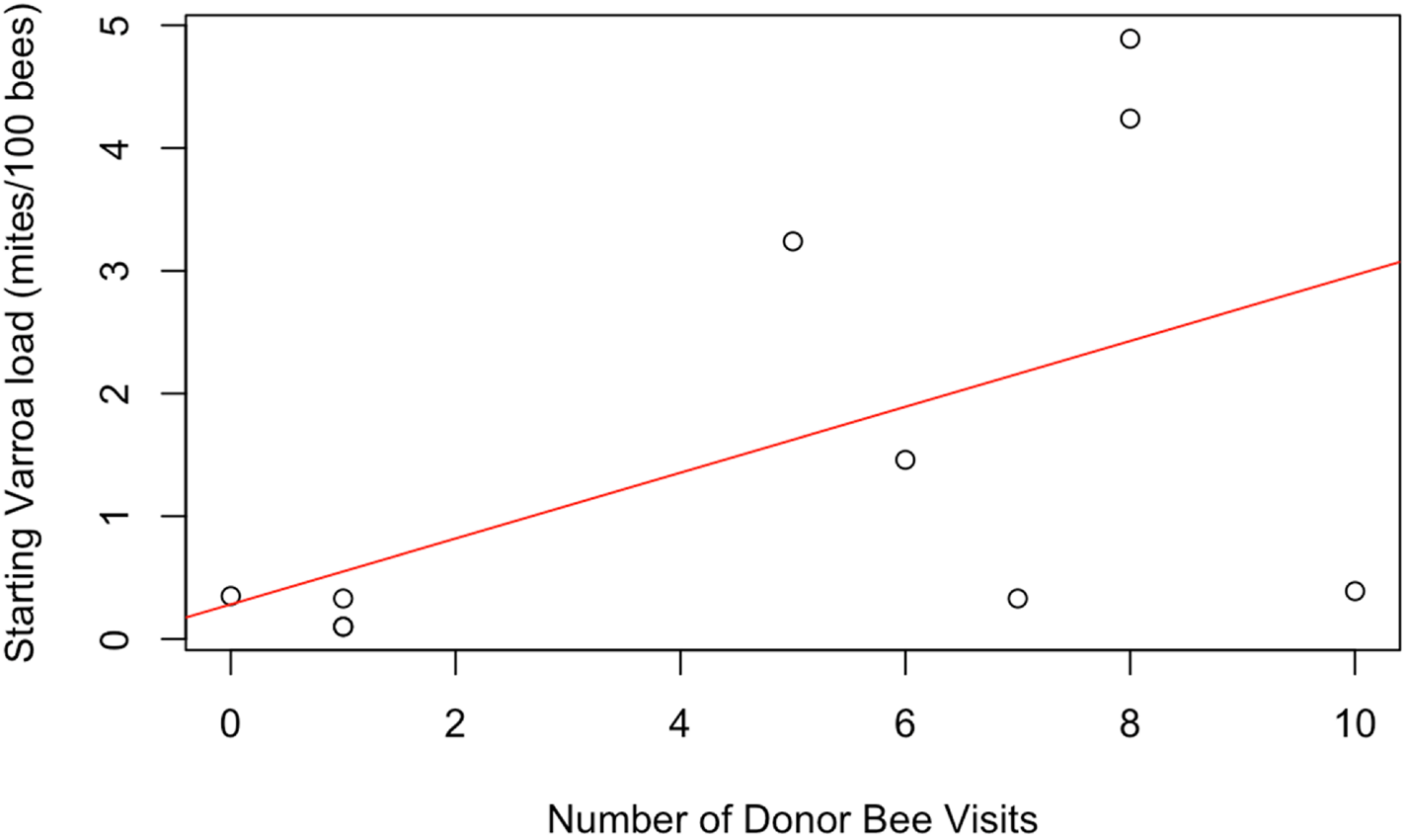
Correlation between a colony's starting Varroa load and the number of donor bee visitors it received. Colonies with higher starting Varroa loads received more visitors than colonies with lower starting Varroa loads (*Spearman's r* = 0.62, *p* = 0.05).

Receiver colonies visited by any donor bee (from high or low mite donor colonies) also started the study with similar mite loads to receiver colonies that were not visited (*t* = 1.34, *df* = 9.33, *p* = 0.21). *Varroa* loads in colonies that were visited by any donor bee, however, increased more than the mite loads in colonies not visited by donor bees (Fig. 12, F1 = 4.57, *p* = 0.03). Within apiaries that were visited by donor bees, there was a positive correlation between a colony’s starting mite load and the number of non-natal bees it received (Fig. 13, Spearman’s *r* = 0.62, *p* = 0.05). There was not a correlation between the number of non-natal bees a colony received and its ending mite load (Spearman’s *r* = -0.14, *p* = 0.71). *Varroa* population growth was associated with whether a colony was visited by non-natal bees or not, and not with the total number of donor bee visitations.

### Robbing screens

**Figure 14:**
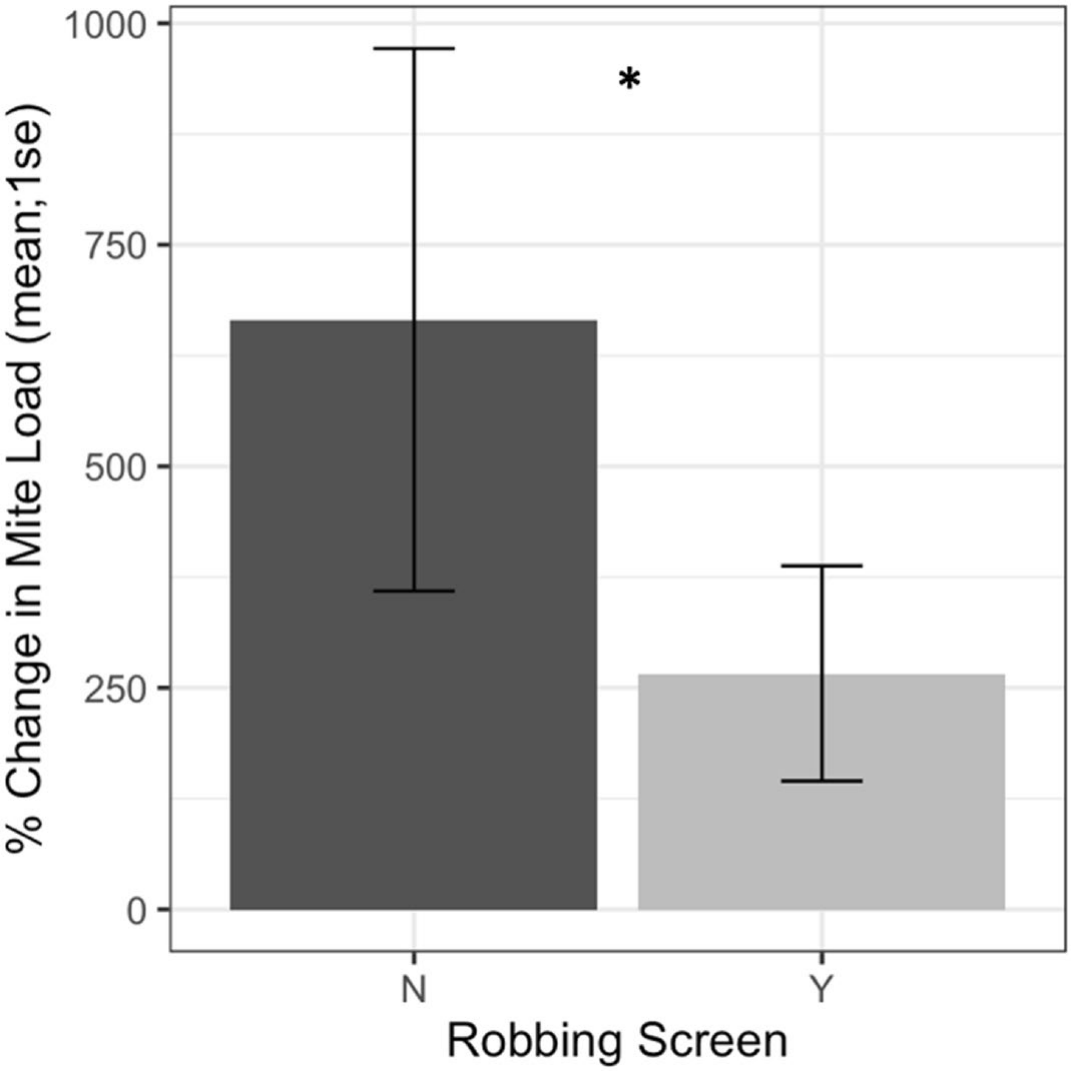
Percent change in Varroa loads in colonies with and without robbing screens. Colonies with robbing screens had reduced increases in Varroa population compared to unscreened colonies (glm *p = 0.02).

**Figure 15:**
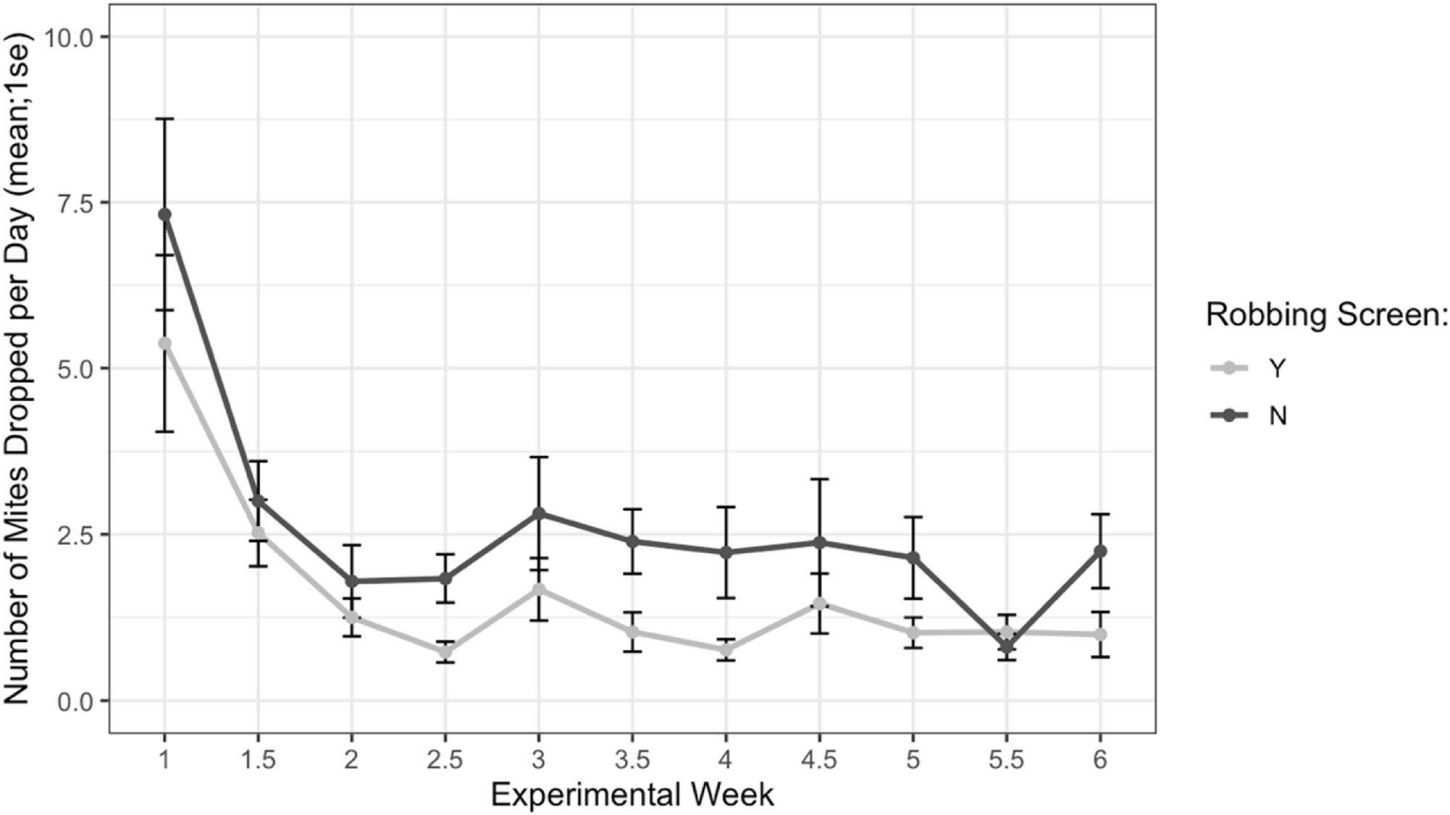
Sticky board Varroa loads in colonies with and without robbing screens over each experimental week. Colonies with robbing screens had fewer mites on sticky boards than colonies without screens (glm ***p < 0.001).

Direct measures of *Varroa* infestation on adult bees from alcohol wash samples showed receiver colonies with and without robbing screens started the experiment at similar infestation levels (*t* = -1.61, *df* = 22.2, *p* = 0.12). During the study, colonies with robbing screens had a lower *Varroa* population growth rate (Fig. 14, F1 = 6.16, *p* = 0.02). Evaluation of the daily *Varroa* drop from colonies via sticky board mite counts show a similar trend, with starting counts not differing between colonies with or without screens (Fig. 15, *t* = -0.99, *df* = 29.8, *p* = 0.33). Sticky board counts over the whole experiment show that colonies with robbing screens had consistently lower *Varroa* loads than colonies without robbing screens (*F*_1_ = 14.31, *p* < 0.001). Sticky board mite counts (mean # of mites dropped per colony per day) in the first experimental week were significantly higher than any other week (first week 6.35 ± 0.98 vs. other weeks 1.71 ± 0.12, t = 4.69, df = 31.9, *p* < 0.001). This is likely due to residual mite drop from the formic acid treatment that ended one day before sticky boards were placed on colonies.

## Discussion

The “mite bomb” hypothesis expects that high mite donor bees will end up in receiver colonies. While high mite colonies systematically lost bees throughout this study, particularly in the last two weeks of the experiment, they were rarely detected at receiver colonies. Instead, the majority of bee visitations to receiver colonies in this study came from low mite donor colonies (Fig. 9), and visitation from high mite donor colonies was not associated with increased rates of *Varroa* population growth (Fig. 11). The “robbing” hypothesis expects that if mites were primarily being brought home on natal bees, robbing screens would not deter these natal bees from returning home, and would thus have no effect on mite population growth. Instead, screened colonies exhibited reduced *Varroa* population growth compared to unscreened colonies (Figs. 14, 15), suggesting that mites are not primarily being brought home on bees returning from robbing high mite colonies. Mites are more likely being transmitted to receiver colonies by non-natal bees, and visitation from any non-natal bee is correlated to accelerated *Varroa* population growth.

These results suggest the need for an alternative hypothesis, which is here referred to as colony permissiveness. Colonies that permit more presumably short term visiting bees experienced greater mite population growth than their less permissive counterparts. The term “visiting” is used here instead of robbing because robbing is typically characterized by mass recruitment of nest mates, so that many non-natal bees are stealing a colony’s resources together^39^. Here, no evidence of recruitment was seen as the number of visitors to each receiver colony was low, thus the visitation behavior observed did not appear as true robbing. It is likely that bees from unmarked colonies were also visiting receiver colonies, and the number of marked visitors detected is probably a small fraction of the real number of non-natal bees visiting receiver colonies. Regardless of the true number of visitors, the motive behind these non-natal bee visitations is unknown, and understanding this behavior may lead to important insights regarding horizontal transmission of mites.

A prior study tracked the movement of bees between high and low mite apiaries and the resulting change in mite populations, and found that colonies exhibited increased mite loads after their bees visited high mite colonies, thus concluding that mite transmission was due to natal bee robbing activity^33^. Both this prior study and the present study, however, observed that high mite colonies are more likely to receive non-natal bees than act as a source of donor bees. The present study further tested the directionality of this phenomenon with the inclusion of robbing screens, finding that screened colonies exhibited reduced *Varroa* population growth. Because robbing screens do not deter natal bees, non-natal visitors are more likely to be a source of mites to the colonies they visit than to bring acquired mites home.

These results provide important clues to understanding the host-parasite relationship of honey bees and the *Varroa*/virus complex. There are two avenues where *Varroa* and/or viral pathogens could alter host behavior to aid in dispersal and transmission. First, elevated *Varroa* and/or viral loads in donor colonies could result in elevated rates of infected bees exiting donor colonies and visiting receiver colonies. These infected non-natal donor bees could transmit both mites and viruses upon entering a receiver colony. Mites can switch phoretic hosts or enter a brood cell within seconds, so even a short visitation from a mite carrying non-natal bee can result in transmission^13,40^. It was also recently demonstrated that bees infected with a *Varroa* vectored virus were more likely to remove themselves from their natal colony, and were more frequently admitted to non-natal colonies^41^. In the present study, high mite donor colonies did not produce more visiting bees than low mite donor colonies. However, viral loads of high and low mite donor colonies are unknown. It is possible that due to their close proximity and the high degree of drift within the donor apiary, low mite colonies experienced elevated viral loads while maintaining low mite loads. It is certainly possible that donor bee behavior was affected by parasite and/or pathogen loads, and further work will attempt to assess to what degree this occurred.

Another potential factor influencing mite population growth is the effect of parasites on colony permissiveness – willingness to accept non-natal bees. Kakugo virus, a close relative of DWV for instance, is found disproportionally in a colony’s guard bees, suggesting some evolutionary advantage to low levels of infection within colonies^42^. Here, robbing screens were used to circumvent a colony’s natural permissiveness, making receiver colonies less accessible. The number of visitations to screened colonies is unknown, but their reduced *Varroa* population growth indicates that they were visited less often. While host-parasite dynamics may affect a colony’s permissiveness, another potentially important factor is the role of long term bee breeding on commercially managed bees. Beekeepers have intentionally selected and propagated gentle bees for generations. Several studies, however, have noted more defensive strains of honey bees often show greater *Varroa* tolerance^43^. It is possible that by breeding gentler bees, beekeepers have inadvertently made colonies more permissive to visits, and thus more susceptible to mite immigration.

Declining colony health or strength either caused by or resulting in elevated mite loads could further affect a colony’s permissiveness. The number of visitors a colony received was positively associated to its starting mite load, indicating that elevated *Varroa* loads can make colonies more permissive to non-natal bee visitation. This finding is supported by prior work which tracked drifting bees and found that high mite colonies were not more likely to produce drifting bees when compared to low mite colonies, but rather high mite colonies were more likely to receive non-natal bees than low mite colonies^34^. Here, a receiver colony’s permissiveness to visitors showed no bias toward low or high mite donor bees, and was associated with an increased mite load. These results suggest that elevated mite or viral loads may alter receiver colony behavior, particularly in guard bees, to admit more non-natal visitors. The importance of receiver colony behavior in this context was unexpected and thus not included in this experiment’s design. While there is evidence that mite loads in these colonies played a role in their permissiveness, other factors that may play a role, such as colony size, were not rigorously measured. As all of receiver colonies started the experiment with an approximately equal number of bees and brood, and all colonies ended with a queen and brood it seems unlikely that colony size played an important role; however, further studies to elucidate the impact of these effects are needed.

These results provide further evidence that mites transmitted between colonies and apiaries on drifting or visiting bees play an important role in driving mite population rates in some colonies. Of interest is that the data supported neither the mite bomb or robbing hypothesis currently suggested as the mechanism by which mites move between colonies. Rather, results suggest that a colony’s permissiveness predicts the rate of mite growth a colony will experience. This finding seems supported by the nascent understanding of the *Varroa*/virus complex and honey bee relationship, hinting at intriguing evolutionary questions regarding host parasite relationships at an individual bee and colony level. Arguably more impactful, however, was the finding that robbing screens, an inexpensive and accessible piece of bee equipment, could dramatically help beekeepers keep mite levels low. While more testing and refinement of this technology is necessary before its use is widely promoted, this offers some much needed hope for an industry struggling with damages caused by *Varroa*.

## Supplementary Information

Supplementary information.

## Data Availability

The datasets generated during and/or analyzed during the current study are available from the corresponding author on reasonable request.
