# Supplementary material for: Accelerated Varroa destructor population growth in honey bee (Apis mellifera) colonies is associated with visitation from non-natal bees
Source: Sci Rep. 2021 Mar 29;11:7092. doi: 10.1038/s41598-021-86558-8 (PMC8007729; doi:10.1038/s41598-021-86558-8)
Supplement: Supplementary file 1 — Supplementary information. [file 41598_2021_86558_MOESM1_ESM.pdf]

Accelerated *Varroa destructor* population growth in honey bee *(Apis mellifera)* colonies is associated with visitation from non-natal bees

Kelly Kulhanek*[1], Andrew Garavito[2], Dennis vanEngelsdorp[2]

Supplementary Information:

Supplementary Table 1. Mite loads in each donor colony at the start and end of the study. These mite loads were counted from samples of 300 adult bees by alcohol wash.

Colony	***Starting Varroa load (mites/100 bees)***	***Ending Varroa load (mites/100 bees)***
Low Mite Colony 1	0.00	2.04
Low Mite Colony 2	0.33	1.85
High Mite Colony 1	4.45	14.69
High Mite Colony 2	14.69	64.22
